# Physical and Psychosocial Factors in the Prevention of Chronic Pain in Older Age

**DOI:** 10.1016/j.jpain.2018.06.001

**Published:** 2018-12

**Authors:** Daisy Fancourt, Andrew Steptoe

**Affiliations:** Department of Behavioural Science and Health, University College London, London, United Kingdom.

**Keywords:** Chronic pain, physical activity, psychosocial, community, positive affect

## Abstract

Chronic pain is recognized as a major challenge as people age. Yet, despite growing research on chronic pain management, there is little research into chronic pain prevention. Thus there is a clear need to identify multimodal activities that could be encouraged among older adults as part of a healthy lifestyle to decrease the incidence risk of chronic pain. Using data from the English Longitudinal Study of Ageing we tracked 2,631 adults aged ≥50 years who were free from chronic pain at baseline across a decade and explore whether physical or psychosocial factors reduced the risk of developing chronic pain. In relation to physical factors, engaging in vigorous weekly activity was protective against the development of chronic pain (odds ratio 0.74, standard error 0.07, 95% confidence interval 0.62–0.89) when controlling for all identified socioeconomic, health, and social confounders. However, no effects were found for moderate weekly activity. In relation to psychosocial factors, cultural engagement was also protective against the development of chronic pain (odds ratio 0.75, standard error 0.07, 95% confidence interval 0.63–0.91), but community group participation was not. These findings extend previous work showing that physical activity and psychosocial factors such as positive affect are key factors in the long-term success of chronic pain self-management. Future interventional studies for chronic pain are encouraged.

**Perspective:**

This article explores whether physical and psychosocial activities could reduce the risk of developing chronic pain in older age. These results could potentially help clinicians to recommend multimodal activities as part of a broader healthy lifestyle for those aged ≥50 years to reduce the incidence rate of chronic pain.

Chronic pain is recognized as a major challenge as people age. Prevalence figures vary, but broadly suggest that around one-half to two-thirds of adults aged ≥50 years are affected (>4 times the number of those 18-25 years of age affected), and incidence rates are as high as 5.4% per year.^7^, ^15^^,^^24^ Pain is one of the most widely cited symptoms of underlying disability in older adults and is linked with impaired physical capacity for basic and instrumental activities of daily living, poorer mobility, and falls.^18^, ^24^^,^^35^ Pain is also associated with psychological outcomes, including fatigue, poor quality of life, and a heightened risk of depression.^10^, ^17^ In addition, it is linked with high economic costs through early retirement, medication, social care needs, and use of health services.^25^ As a result, it is important to identify ways of reducing the risk of developing chronic pain. However, to date, there has been little research into this topic. A 2016 systematic review identified 9 published reports on physical activity for the prevention of chronic pain.^30^ Physical activity has been found to decrease the incidence of chronic low back pain in 7 of these reports, with data suggesting that intensive programs are required for effects to emerge.^20^, ^28^ However, these studies have involved relatively small sample sizes (only 3 with a sample size of >100) and a range of ages, and so do not provide a clear picture about strategies for reduction of chronic pain incidence in older adults, when it is most common. Relatedly, having a high body mass index is a predictor of chronic pain^34^ and a sedentary lifestyle has been proposed as another predictor.^32^ However, other studies have reported no significant associations between physical activity and chronic pain incidence, so our understanding of physical activity as a potential risk-reducing factor for chronic pain remains underdeveloped.^4^ Alongside physical predictors, there is increasing recognition of psychosocial factors in chronic pain. As well as being an outcome of chronic pain, affective factors such as depression also predict future pain, as do anxiety disorders, stress, negative thoughts, neuroticism, and catastrophizing.^12^, ^14^^,^^19^, ^26^^,^^29^ Broader social factors such as low perceived social acceptance and poor social relations have also been identified as predictors.^14^, ^32^ In addition, there is evidence to suggest that cognitive interpretation of pain is key to its perception and classification as chronic pain, with high levels of self-efficacy and coping resources seeming to be protective.^33^

Consequently, it seems that combinations of physical and psychosocial factors could be protective against the development of chronic pain in older age. However, there remains a clear need to identify specific multimodal activities that could be encouraged as part of a broader healthy lifestyle. This multimodal approach is recognized and practiced within chronic pain management, but is less researched in chronic pain prevention.^1^ Therefore this article explored longitudinal associations between physical and psychosocial activities and chronic pain incidence in older age, among individuals initially free of chronic pain. For physical activities, we focused on both moderate and vigorous physical activity to ascertain whether there are specific benefits independent of the avoidance of a sedentary lifestyle. For psychosocial activities, a conceptual model for the relationship between positive affect and chronic pain has been proposed, which posits that positive affect and related emotional responses elicited through engagement in stimulating and novel experiences can buffer perceptions of pain, buffer the effect of pain on physical function, increase daily positive interpersonal events that themselves buffer perceptions of daily pain, foster mindfulness as a means of disengaging from cognitive fixation on pain, broaden the scope of attention to encompass nonpainful stimuli, and support positive reappraisal and adaptive coping.^8^ These factors are not only helpful for the management of chronic pain but can also stop initial pain from developing into a chronic problem, thereby playing a role in its prevention. There is increasing research focusing on the health and well-being benefits of psychosocial activities such as participation in community groups and cultural engagement,^2^, ^3^ so we focused on these 2 psychosocial activities to ascertain whether they are protective against the development of chronic pain.

## Methods

### Participants

We used data from the longitudinal cohort study the English Longitudinal Study of Ageing. The English Longitudinal Study of Ageing contains a representative sample of adults aged ≥50 years of age living in England.^18^ We specifically worked with data from wave 2 (2004/05) across every biennial wave through to wave 7 (2014/2015) for a total of 6 waves and a decade of data. The study received ethical approval from the National Research Ethics Service and all participants provided informed consent.

### Measures

We measured pain using participant self-report using the questions, “Are you often troubled with pain?” and, if so, “How bad is the pain most of the time?” (with options of mild, moderate, or severe). In line with previous research, we specifically focused on pain that was classed as moderate or severe,^35^ and an index was created of whether participants reported such pain at any point across the 10 years (our chronic pain incidence index). Participants were additionally asked to specify where they felt their pain occurred: all over versus a specific common site (back, hips, or knees) versus other.

Our exposures were all measured at baseline (wave 2). Specifically, we measured 2 types of physical activity: the frequency with which participants took part in “sports or activities that are moderately energetic” or “vigorous” (hardly ever or never, 1–3 times a month, once a week, >1 time per week). Owing to marked skew across these 2 variables (negative skew for moderate activity and positive skew for vigorous activity), responses were dichotomized into once a week or more versus less frequently. We measured 2 types of psychosocial activities: frequency of engagement with community groups (including political parties, trade unions, environmental groups, tenants/residents associations, neighborhood watch, church or religious groups, charitable associations, evening classes, social clubs, sports clubs, exercise classes or other clubs/societies) and frequency of engagement with cultural activities (including going to museums, art galleries, exhibitions, concerts, the theater, or the opera). Both psychosocial activities were dichotomized into every other month or more versus less frequently.

Factors identified as predicting both physical and psychosocial engagement and chronic pain incidence were included as covariates used in the analysis: age (in years), gender (male or female), ethnicity (white British vs other), educational qualifications (no educational qualifications, O-levels, A-levels, degree/higher or equivalent), total nonpension wealth (quintiles),^22^ living with a spouse or partner (rather than alone), employment status (working full time vs working part time vs not working), presence of a longstanding physical illness (including cancer, chronic obstructive pulmonary disease, diabetes, angina, or a stroke in the last 2 years), whether participants had arthritis, frequency of alcohol consumption (1–2 days a week, 3–4 days a week, 5*–*6 days a week, or daily), whether participants currently smoked, depression (using the Centre for Epidemiological Studies Depression scale), whether participants had experienced restless sleep in the past week, whether participants were sedentary (doing mild activity less than twice a week), and whether participants were socially isolated (meeting, telephone, or emailing family or friends less than once a week).

### Statistical Analysis

A total of 3,470 participants provided data across all waves for exposure and outcome variables. A total of 112 participants (3.1%) provided incomplete data on covariates so were excluded from analyses (ethnicity, n = 1; employment, n = 24; wealth, n = 47; depression, n = 3; and alcohol consumption, n = 34). We further excluded 3 participants who were registered as blind providing a final sample size of 3,358. Owing to the possibility of left censoring, whereby participants could enter the study having had chronic pain for many years and have different profiles of physical and psychosocial activities, we excluded all participants with chronic pain at baseline (n = 727).

We used one-way analyses of variance and χ^2^ tests to explore demographic differences between those who did and did not report chronic pain across the decade. We then used logistic regression analyses to calculate the odds ratio (OR) of developing chronic pain depending on our physical and psychosocial exposures. Our analyses adjusted for all identified confounders (age, gender, ethnicity, educational qualifications, wealth, cohabitation, employment, physical illnesses, arthritis, alcohol consumption, depression, sedentary behaviors, and social isolation). We also carried out 3 sets of sensitivity analyses. First, to ascertain whether reported chronic pain was indeed chronic rather than of a limited duration, we calculated the number of occasions across the biennial waves of data collection on which participants reported chronic pain, and reran analyses only counting pain as chronic if it was reported across ≥2 waves as per previous protocols.^35^ Second, we explored whether there were differences depending on whether participants reported pain at specific sites (back, hips, or knees) or all-over, generalized pain by rerunning the analyses with specific chronic pain and generalized chronic pain as the outcome measure. Third, we carried out 2 sensitivity analyses to guard against the possibility of reverse causation. We explored our analyses excluding those who experienced even mild pain at baseline (n = 1,114), in case this mild pain was a precursor to more severe pain and was already affecting their participation in activities. And we also reran our analyses excluding those who developed chronic pain in the first wave after baseline (n = 348) and those who had preexisting chronic conditions (n = 213) or arthritis (n = 910) at baseline in case physical challenges preceding the development of pain were affecting activity engagement. Finally, we reran our analyses including mild pain in our definition of chronic pain. This decreased the sample size who were pain free at baseline to 2,244 and led to a 54.2% incidence rate across the 10 years.

All regression assumptions were met. We weighted all data using baseline cross-sectional weights derived from the English Longitudinal Study of Ageing to ensure the sample was representative of the English population and to account for differential nonresponse across the following 10 years based on demographic predictors. All analyses were carried out using STATA version 14 (StataCorp, LLC, College Station, TX).

## Results

### Demographic Characteristics

Our sample consisted of 53.4% women, with an average age of 63.0 years (standard deviation [SD] 7.7 years) at baseline, increasing to 72.5 years (SD 7.0 years) 10 years later. Participants were predominantly white (99.1%), 74.5% were cohabiting with a partner, 36.6% worked part time or full time, 4.6% of participants reported ≥1 chronic conditions, and 18.2% reported having arthritis. In addition, 8.7% showed above-threshold symptoms of depression.

At baseline, 88.3% of participants reported doing moderate activities or sports on a weekly basis or more, and 39.1% reported doing weekly vigorous activities or sports. There were 37.7% who reported engaging with culture every few months or more and 23.0% who reported taking part in community groups.

Across the 10 years, 1,119 participants (42.5%) experienced moderate to severe chronic pain and for 587 participants (22.3%) this moderate to severe chronic pain was experienced on ≥2 waves. A total of 895 participants (34.0%) reported localized pain in one or more of the back, hips, knees, or feet, whereas just 62 participants (5.5%) reported their pain to be all over and the remaining classed their pain as other.

Women were more likely to report chronic pain over the decade, as were those who were not cohabiting with a partner, those with lower educational attainment, those with lower wealth, those who no longer worked, those with a chronic health condition, those with arthritis, those with depression, those experiencing restless sleep, those who consumed less alcohol, and those who met up socially more often. Notably, there were significant differences between levels of sedentary behaviors or smoking behaviors and chronic pain incidence (Table 1).

**Table 1:** Participant Demographics at Baseline Split by Whether Participants Developed Chronic Pain Over the Following 10 Years

	Participants Who Do Not Develop Chronic Pain (n = 1,512)	Participants Who Develop Chronic Pain (n = 1,119)	*P*
Age, mean ± SD	62.6 ± 7.6	63.5 ± 7.8	**.004**^a^
Female	48.4	60.1	**<.001**
White	98.9	99.3	.29
Married/cohabiting	77.2	70.9	**<.001**
Educational attainment			**<.001**
No qualifications	22.0	33.5	
O-level/GCSE	22.5	20.9	
A-level	32.9	31.6	
Degree	22.6	13.9	
Employment			**<.001**
Not working	48.7	59.8	
Working part time	20.6	17.7	
Working full time	30.7	22.5	
Wealth quintiles			**<.001**
1 (lowest)	13.1	20.1	
2	17.6	21.1	
3	21.0	20.6	
4	22.4	19.7	
5 (highest)	25.9	18.5	
Existing chronic condition	3.6	6.1	**.003**
Existing arthritis	10.0	29.3	**<.001**
Existing depression	6.0	12.3	**<.001**
Frequency of alcohol intake			**.001**
1-2 days a week	16.5	22.3	
3-4 days a week	11.7	11.4	
5-6 days a week	42.5	40.7	
Nearly every day	29.3	25.6	
Current smoker	11.1	11.2	.96
Experiencing restless sleep	29.4	42.9	**<.001**
Sedentary lifestyle (mild activity less than twice weekly)	15.3	13.8	.26
Socially isolated (social contact <less than once a week)	25.7	21.0	**.005**

Values are percent unless otherwise noted.

NOTE. All tests show χ^2^ test results, with the exception of^a^^,^ which used a 1-way analysis of variance.

### Physical and Psychosocial Engagement and Pain Incidence

**Figure 1:**
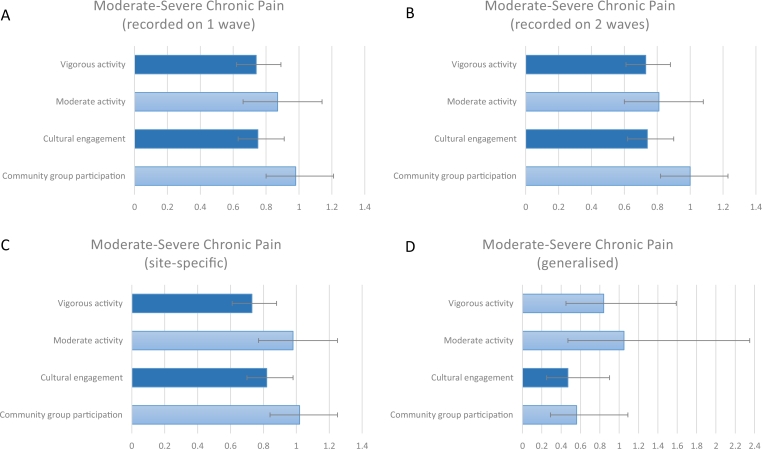
Associations between physical and psychosocial factors and chronic the development of chronic pain over the following decade in adults aged ≥50 years. ORs and confidence intervals. All results are adjusted for age, gender, ethnicity, educational qualifications, wealth, cohabitation, employment, physical illnesses, arthritis, alcohol consumption, smoking, depression, sleep quality, sedentary behaviors, and social isolation.

In terms of physical factors, engaging in vigorous weekly activity was protective against the development of chronic pain across a decade (OR 0.74; standard error [SE] 0.07, 95% confidence interval [CI] 0.62–0.89). However, no effects were found for moderate weekly activity. Of the psychosocial factors, cultural engagement was also protective against the development of chronic pain (OR 0.75, SE 0.07, 95% CI 0.63–0.91) after adjustment for all covariates. However, no effects were found for community group participation (Fig 1A).

### Sensitivity Analyses

Our first sensitivity analysis tested whether the pain was indeed chronic through reanalyzing the data looking for chronic pain reporting on ≥2 waves. Our results were unchanged; in terms of physical factors, engaging in vigorous weekly activity was protective against the development of chronic pain across a decade (OR 0.73, SE 0.08, 95% CI 0.58–0.91). However, no effects were found for moderate weekly activity. Cultural engagement was also protective against the development of chronic pain (OR 0.74, SE 0.09, 95% CI 0.59–0.93). However, no effects were found for community group participation (Fig 1B).

Our second sensitivity analysis explored whether there were differences depending on whether participants reported localized or generalized pain. For localized pain, engaging in vigorous weekly activity was associated with a reduced risk (OR 0.73, SE 0.07, 95% CI 0.61–0.88), whereas no effects were found for moderate weekly activity. Cultural engagement was also protective (OR 0.82, SE 0.08, 95% CI 0.68–0.999), with no effects for community group participation (Fig 1C). For all-over generalized pain, neither moderate nor vigorous activity was associated with a reduced risk of pain incidence (although it should be noted that only 62 participants reported generalized chronic pain over the decade). However, cultural engagement was associated with a reduced risk (OR 0.47, SE 0.15 95% CI 0.25-0.90). Community group participation was not significant (incidence risk ratio 0.58, SE 0.18, 95% CI 0.31–1.08; Fig 1D).

Our third sensitivity analysis exploring the possibility of reverse causation did not materially affect results. When excluding participants with mild pain at baseline, engaging in vigorous weekly activity was still associated with a reduced risk of pain (OR 0.70, SE 0.07, 95% CI 0.57–0.85), whereas no effects were found for moderate weekly activity. In addition, cultural engagement was also still protective (OR 0.78, SE 0.08, 95% CI 0.64–0.96), with no effects for community group participation. When excluding participants who developed chronic pain in the first wave after baseline, engaging in vigorous weekly activity was still associated with a reduced risk (OR 0.75, SE 0.08, 95% CI 0.61–0.91), whereas no effects were found for moderate weekly activity. Cultural engagement was also still protective (OR 0.80, SE 0.08, 95% CI 0.65–0.98), with no effects for community group participation. Similarly, when excluding participants with either chronic health conditions at baseline or arthritis at baseline, the significance of results was not affected. Finally, when expanding our definition of chronic pain to include incident mild pain, we found the same pattern of results, with just vigorous weekly activity (OR 0.79, SE 0.07, 95% CI 0.66–0.96) and cultural engagement (OR 0.80, SE 0.08, 95% CI 0.65–0.97) associated with a lower risk of developing chronic pain.

## Discussion

This study is the first to explore simultaneously potential physical and psychosocial protective factors for the development of chronic pain in older adults. Our results demonstrate that both vigorous weekly activity and regular cultural engagement seem to reduce risk of incident chronic moderate to severe pain, recurring chronic pain, and site-specific pain. Cultural engagement also seems to be a risk-reducing factor for the development of widespread pain. These associations were found independent of demographic, socioeconomic, and health-related covariates, as well as sedentary behaviors and social isolation.

Although there are few existing studies on the prevention of chronic pain incidence, our findings echo previous findings and related literature. First, it is notable that vigorous but not moderate physical activity was associated with a lower pain incidence. Previously, intensive physical activity (daily stretching and twice weekly muscle endurance training) has been found to decrease the incidence of low back over 12 months,^28^ but moderate physical activity (daily muscle strength exercises) does not seem to decrease the incidence of low back pain in adults over 24 months.^11^ Therefore this study reinforces the suggestion that only vigorous activity is protective against the development of chronic pain. However, it is notable that both previous studies have been with working age adults rather than older adults and are therefore not directly comparable. Older adults may have greater endogenous pain facilitation and a reduced capacity to endogenously inhibit pain, which increases their risk of chronic pain.^22^ Therefore older adults need to be studied separately from younger adults. In considering why vigorous activity was found to be a risk-reducing factor for chronic pain in this study, there are several hypotheses. First, chronic low-grade systemic inflammation and oxidative stress become more common as adults age, both of which have been demonstrated to induce sensitization of pain pathways.^9^, ^16^^,^^23^, ^36^ Vigorous physical activity has been found to have anti-inflammatory and antioxidant effects that could reduce this sensitization. Second, vigorous physical activity may be associated with lower temporal summation of pain in older adults; a finding not seen for lighter physical activity.^22^ Temporal summation is often used as an indirect marker of central sensitization to pain. Therefore regular vigorous (but not moderate) physical activity may be protective against the development of chronic pain in older age.

This study also found evidence that psychosocial factors may be protective against the development of chronic pain, in particular engagement in cultural activities such as going to museums, art galleries, exhibitions, concerts, the theater, or the opera. It is notable that the ORs for cultural engagement were directly comparable with those of vigorous physical activity, suggesting a reduction of 25% to 26% in risk of chronic pain incidence. In considering why these effects emerged, cultural engagement is a multimodal activity, composed of social engagement, gentle physical activity, and positive affect responses. In this study, the benefits of cultural engagement were found independent of sedentary behaviors, physical activity, and social isolation, suggesting that these components in themselves are not responsible for the associations noted. However, other studies have identified psychological benefits of cultural engagement, including the enhancement of well-being, the prevention of the development of depression, and the recovery from mental illness.^5^, ^21^ Notably, these positive psychological benefits have not been found consistently for community group membership,^6^ which could explain the differences in association with chronic pain found in this study. Indeed, it is notable that, for participants who experienced widespread pain, only psychosocial factors and not physical factors were found to be risk reducing. Patients with widespread pain typically report high levels of stress, anxiety, and depression and the condition is often comorbid with psychiatric disorders.^27^, ^31^ As a result, psychosocial activities may be particularly effective for these patients.

Because this study is observational rather than experimental, causality cannot be assumed. However, we used a large and nationally representative sample of older adults in England, tracked reports of pain over a decade, controlled for all identified confounding variables, and ran a series of sensitivity analyses testing potential reverse causality. Although it is possible that latent confounding factors may explain our results, it is notable that we found results for some but not all factors (such as vigorous but not moderate physical activity), which have the same associated confounding variables. In noting that this sample was nationally representative for Britain, it is relevant that the sample was largely white British, and as such whether the results can be generalized to different ethnicities remains unknown. Finally, our outcome measure was self-reported rather than based on an objective physical test, and the interview asked participants if they were often troubled by pain, rather than giving a specific timeframe to consider. However, we tested our definition of chronic pain through a several sensitivity of analyses, which confirmed our initial findings.

## Conclusions

This study supports previous work suggesting that vigorous (but not moderate) physical activity can be protective against the development of chronic pain in older age and showed for the first time that cultural engagement could be a protective psychosocial factor. These findings extend previous work showing that patient motivation, coping, self-efficacy, and positive affect are key factors in the long-term success of chronic pain self-management^13^ to suggest that such factors could also be encouraged through psychosocial interventions such as cultural engagement to reduce chronic pain incidence, especially in those with widespread pain. This study has implications for clinicians working with patients with chronic pain because it suggests that vigorous physical activity can be recommended to help prevent chronic pain; a recommendation that ties in with other well-known health benefits of physical activity. It also suggests that schemes such as social prescribing of cultural activities could be of value to help prevent the development of chronic pain.
